# Granulocytic sarcoma of the breast without development of bone marrow involvement: a case report

**DOI:** 10.1186/1746-1596-4-2

**Published:** 2009-01-06

**Authors:** Teresa A Vela-Chávez, Myrna D Arrecillas-Zamora, L Yolanda Quintero-Cuadra, Falko Fend

**Affiliations:** 1 Department of Pathology, Instituto Nacional de Cancerología, Av. San Fernando 22, Col. Sección XVI, Del. Tlalpan, CP 14080, Mexico City, Mexico; 2 Department of Pathology, University of Tuebingen, Liebermeisterstr. 8, D-72076, Tuebingen, Germany

## Abstract

A 29-year-old woman presented with a breast tumor with a primary diagnosis of MALT lymphoma. A repeat biopsy revealed a hematological neoplasm with diffuse, Indian file, and targetoid patterns. The cells were intermediate size with eosinophilic granules; the immunophenotyping showed monocytic differentiation, and no lymphoepithelial lesion was observed. The diagnosis was granulocytic sarcoma. Three different bone marrow biopsies were negative for neoplastic infiltration. After treatment, she developed secondary pancytopenia which contributed to her death 16 months after primary diagnosis. Granulocytic sarcoma of the breast is uncommon. A complete panel of immunohistochemistry is necessary to perform this diagnosis.

## Background

Granulocytic (myeloid) sarcoma (GS) is a rare hematological neoplasm composed of immature myeloid cells at an extramedullary site which may precede leukemia; nevertheless, the most frequent clinical presentation is secondary to acute or chronic myeloid leukemia, or myelodysplastic syndrome [1,2] developing within an average of 6–12 months after primary diagnosis [3].

The most common sites of presentation of GS are bone, lymph nodes, soft tissues, and skin; involvement of breast is uncommon [4-6]. The majority of the cases have been associated with synchronous or metachronous leukemia. However, in few published cases, no further disease manifestations developed [1,3,7].

Patients with GS have a poor prognosis, and the majority of patients without bone marrow infiltration at presentation die of leukemia within an average of 16.5 months after diagnosis [2,3].

GS of the breast presents a pattern of infiltration similar to lobular carcinoma or lymphoma [6-8], as which it is frequently misdiagnosed.

We report the case of a patient with GS involving the breast with no evidence of a myeloproliferative disease during the subsequent 16 months, and histological and immunophenotyping features are needed in order to avoid misdiagnosis.

## Case presentation

A 29-year-old woman presented with a three month history of a palpable breast tumor of 3 cm of diameter on the right side. She underwent a breast biopsy and a primary diagnosis of MALT type non-Hodgkin lymphoma was rendered in another institution; subsequently, the patient was treated with 3 cycles of CHOP chemotherapy without improvement. Three months later, she presented in our institution with increase of the tumor to 5 × 4 cm. The peripheral blood showed a white blood cell count of 5.2 G/l, and Hb 12.7 g/l. No blasts cells were identified in the peripheral blood smear or in bone marrow trephine. She underwent a radical mastectomy, and after diagnosis, the patient was treated with radiotherapy (30 Gy to the axillary area with photons and 25 Gy to the thoracic wall with electrons 15 MeV).

Four months after mastectomy, the tumor relapsed in the eyelid, abdominal wall, with additional soft tissue infiltration of the thighs as well as lymph nodes of the left groin. A repeat bone marrow biopsy was performed and showed no neoplastic infiltration, and fluorescence in situ hybridization failed to demonstrate a *BCR/ABL *translocation. She received chemotherapy for acute myeloid leukemia according to the 7+3 scheme with Ara-C (163 mg/day) and daunorubicin (32 mg/day). Subsequently, she received radiotherapy and chemotherapy with higher dose of cytarabin (HIDAC). After treatment, the patient developed pancytopenia, resulting in hemorrhagic diathesis (echimosis, petechiae and gingivorrhagia) and pneumonia. The bone marrow trephine remained negative for infiltration. The patient developed intracranial hemorrhage corroborated by computed tomography, resulting in deep coma. She expired 16 months after primary diagnosis.

### Histological and immunohistochemical findings

**Figure 1:**
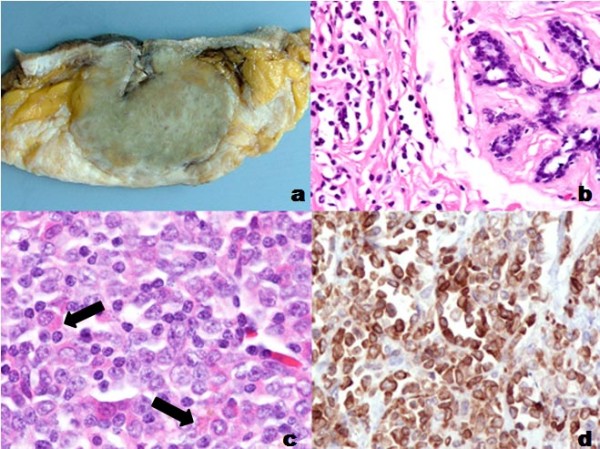
**a) Gross appearance, cut surface solid, green, firm, and well-circumscribed**. b) Neoplastic cells are surrounding without involvement of duct or lobular structures (H&E 100×). c) The cells are of intermediate size with scant cytoplasm, irregular nuclei, clumped chromatin, and small nucleoli; some cells contain eosinophilic granules (H&E 400×). d) Myeloperoxidase reactivity is intensively positive in neoplastic cells (400×).

The assessment of the breast biopsy performed at our institution showed an infiltrative neoplasm, mainly with a diffuse pattern that alternated with Indian file or targetoid pattern. The cells were of intermediate size with scant cytoplasm, irregular nuclei, clumped chromatin and small nucleoli; some cells contained eosinophilic granules. Epithelial structures, including ducts and lobules, were preserved with surrounding neoplastic cells (targetoid pattern). Lymphoepithelial lesions were not identified, not even with immunohistochemical studies (Figure 1B, 1C). Naphtol AS-D chloroacetate esterase was strongly positive, and mucin stains were negative.

The neoplastic cells showed strong immunoreactivity for CD68 (KP1), myeloperoxidase, CD34, CD117, CD43, and lysozyme (Figure 1D); CD45 showed weak staining. CD3, CD20, CD10, CD15, CD56, CD68 PGM1, bcl-2 and TdT were negative, as well as epithelial markers (epithelial membrane antigen, cytokeratin AE1/AE3). The final diagnosis was granulocytic sarcoma of the breast, with some features of monoblastic differentiation, as evidenced by strong lysozyme expression.

The mastectomy specimen revealed a well-demarcated tumor measuring 5 × 4 cm, of green color and increased consistency (Figure 1A). The histological findings were similar to the biopsy, the axillary dissection showed partial involvement of two lymph nodes. Three repeat bone marrow biopsies were performed at different times without evidence of neoplastic infiltration.

## Discussion

GS of the breast is an unusual site of presentation occurring mainly in young women, with high incidence of bilaterality [1-3,5]. Mainly, the patients present the breast engorgement, and a painless mass without associated local symptoms [1]. In this case, the patient developed a rapidly growing, non-tender tumor, affecting only the right breast without bone marrow involvement.

GS of breast shows different patterns of infiltration: diffuse, Indian file, targetoid and starry sky pattern [1]. The differential diagnosis includes non-Hodgkin lymphoma (predominantly MALT lymphoma and diffuse large B-cell lymphoma), lobular carcinoma and small round cell tumors [2,4,9]. Inflammation and extramedullary hematopoiesis enter into the differential diagnosis when the maturation of the cells is prominent [10].

In this case, the primary diagnosis was MALT lymphoma, but the presence of granules, lack of lymphoepithelial lesions and positivity of granulocytic markers rule out this possibility [1,4,11].

Lobular invasive carcinoma is the second most frequent neoplasm of the breast; misdiagnosis is frequent because GS can present with the same pattern of infiltration. The presence of carcinoma in situ, droplets of mucin, and loss of cohesiveness are some histological features to exclude a GS diagnosis [12], as well as the positivity for epithelial markers or mucin stains [1,4,11].

A complete panel of immunohistochemistry is helpful to recognize this entity with an isolated presentation. GS is immunoreactive for myeloperoxidase, CD117, and CD68, CD43 is positive in most of the cases, and 75% are reactive for CD45 [10,13]. Lysozyme and CD68 PGM1 expression are evidence for monocytic differentiation.

Generally, patients with GS develop a myeloproliferative disease within 6–12 months, nevertheless, in this case the patient never presented with myeloproliferative or myelodysplastic disease in bone marrow biopsies, and the peripheral blood never showed blasts during the 16 months. This is an uncommon phenomenon reported in few cases [1,3,5,7-9,14-16]. Due to the rarity of isolated GS and different treatments, the clinical outcome and the prognosis in this group of patients is hard to predict. In our patient, the use of the HIDAC regimen, which shows significant myelotoxicity, lead to severe pancytopenia which contributed to her death.

## Conclusion

GS is an uncommon breast neoplasm, and the clinical presentation without development of a myeloproliferative disorder is extremely rare. Ancillary studies are necessary to recognize this entity.

## Abbreviations

GS: granulocytic sarcoma; MALT: mucosa associated lymphoid tissue

## Consent

Written informed consent was obtained from the patient for publication of this case report and accompanying images. A copy of the written consent is available for review by the Editor-in-Chief of this journal.

## Competing interests

The authors declare that they have no competing interests.

## Authors' contributions

TV-C participated in conception of the idea, writing of the manuscript, and interpretation of histological assays. MA-Z carried out the immunoassays, collected data, and revised the manuscript. YQ-C participated in interpretation of biopsies, review of the literature, and writing of the manuscript. FF revised the manuscript critically for important intellectual content, and gave final approval of the version to be published.
